# Effects of 6-Week Traditional and Functional Resistance Training on Arterial Stiffness and Muscular Strength in Healthy Young Men

**DOI:** 10.3389/fphys.2022.859402

**Published:** 2022-03-02

**Authors:** Chongwen Zuo, Qing Li, Li Zhang, Shumin Bo

**Affiliations:** ^1^Graduate Department, Capital University of Physical Education and Sports, Beijing, China; ^2^School of Kinesiology and Health, Capital University of Physical Education and Sports, Beijing, China

**Keywords:** resistance training, arterial stiffness, vascular health, cardio-ankle vascular index, functional resistance training

## Abstract

**Background:**

The present study investigated the effects of traditional resistance training (TRT) and functional resistance training (FRT) on arterial stiffness and muscular strength in healthy young men.

**Methods:**

This randomized controlled trial included 29 untrained healthy young men aged 18–29 years who were randomly divided into two groups, namely, TRT group (*n* = 15) and FRT group (*n* = 14). All participants underwent numerous tests, such as those for body composition, cardio-ankle vascular index, blood pressure, heart rate, and maximal strength before and after the 6-week training program. The exercise training comprised whole-body strength training exercises 3 days a week for 6 weeks. The total training volume and number of sets (4–5 sets) were kept constantly similar in each group. The TRT group completed 4–5 sets of 8–12 repetitions [70% of 1 repetition maximum (1RM)], whereas the FRT group completed 4–5 sets of 15–22 repetitions (40% 1RM).

**Results:**

The TRT and FRT groups exhibited equally significantly increased maximal strength (within group: both *p* < 0.01). Furthermore, the independent *t*-test showed that the differences between the two groups in terms of changes in maximal strength were no significant (between group: both *p* > 0.05). Additionally, significant main effects of time (pre vs. post) were observed for the left and right cardio-ankle vascular indices (*p* < 0.05); however, no significant difference were observed between the groups. For body compositions outcome measures, no significant differences between groups were observed.

**Conclusion:**

Six weeks of FRT and TRT exhibit no difference in terms of effects on arterial stiffness and muscular strength.

## Introduction

Cardiovascular disease is a major public health problem. In particular, inactivity is associated with thickening, deformation, or hardening of the arterial wall, all of which eventually lead to an increase in the arterial stiffness, systolic blood pressure, and pulse pressure and decrease in the arterial buffering function and blood flow. Age-related growth in arterial stiffness also contributes to an increased risk of heart disease, hypertension, and coronary ischemic disease (Tanaka et al., 1998; Liao et al., 1999; Alan et al., 2003). Additionally, arterial stiffness is a strong independent predictor of cardiovascular events and mortality (Laurent et al., 2001). Studies have identified and characterized the stimuli that accelerate arterial stiffness, such as smoking (Thitiwuthikiat et al., 2017), alcohol (Gonzalez-Sanchez et al., 2020), insufficient sleep (Chami et al., 2016), inactivity, and sedentariness (O’Donovan et al., 2014). Thus, the prevention of cardiovascular events has become a crucial strategy for cardiovascular disease diagnosis and treatment. The American College of Sports Medicine and the American Heart Association have recommended regular aerobic exercise and resistance training as effective conservative methods for the prevention and treatment of cardiovascular disease and aging-related frailty (Williams et al., 2007; American College of Sports Medicine et al., 2018).

Regular aerobic exercise has been reported to inhibit the aging-related increase in arterial stiffness and attenuate the incidence of cardiovascular disease in healthy adults (Siasos et al., 2016; Sardeli et al., 2018). However, the relationship between resistance training and arterial stiffness progression remains to be ascertained (Li et al., 2015b; Evans et al., 2018). A cross-sectional study indicated that young men with resistance training experience have stiffer peripheral arteries than their sedentary peers (Miyachi et al., 2003). Additionally, a study reported that resistance training is associated with high systolic pressure, leading to high stress on the arterial walls, and it could be the major factor for an increase in arterial stiffness (Johnson, 2001). This finding indicates that resistance training may have some adverse effects on arteries. Specifically, some studies have reported that the young and middle-aged individuals who underwent traditional resistance training (TRT; at least three times per week) demonstrated higher levels of arterial stiffness than their inactive peers (Miyachi et al., 2004; Cortez-Cooper et al., 2005; Rakobowchuk et al., 2005; Okamoto et al., 2006). Evidence also suggests that resistance training may not have any unfavorable effect on arterial stiffness in middle-aged adults (Cortez-Cooper et al., 2008; Yoshizawa et al., 2009). Moreover, a meta-analysis (Ceciliato et al., 2020) and two randomized control trials (Casey et al., 2007; Werner et al., 2019) have reported that long-term resistance training (average threee times per week) causes no changes in arterial stiffness among healthy young individuals (average age, 29). Although TRT is effective in improving skeletal muscle mass and function and reversing osteoporosis (Pollock et al., 2000), further studies are warranted to ascertain its effect on arterial stiffness.

Strategies to avoid arterial stiffness can prevent aging-related cardiovascular events. Sports exercise has been recognized as a non-drug method for preventing arterial stiffness by increasing number of young individuals. Functional resistance training (FRT) has been reported to elicit physical capabilities (muscle mass, strength, and dynamic balance) and physical performance similar to traditional resistance training, although with a lower training intensity (Vasconcelos et al., 2020; de Resende-Neto et al., 2021). Nevertheless, the overall influence of FRT on age-associated arterial stiffness is poorly understood. Functional resistance training involves synchronized, multidimensional, and multiple joint movement modes to train muscles with dynamic exercises and continuous changes on unstable surfaces (e.g., BOSU ball, swish ball, and balance disc) for enhancing physical fitness (Feito et al., 2018), and it has been applied to improve the physical condition and health of players and for the postoperative recovery of patients and young adults (Kibele and Behm, 2009; Xiao et al., 2021). Additionally, FRT is the most promising method for promoting multisystemic adaptations because it uses resistance training not only for central body strength gains but also for multiple capabilities (e.g., coordination, agility, flexibility, and cardiorespiratory endurance; La Scala Teixeira et al., 2017; de Resende-Neto et al., 2019b). However, the effect of FRT with unstable surface resistance training on arterial stiffness has not yet been demonstrated. The present study attempted to investigate the effects of 6-week TRT and FRT programs on arterial stiffness and muscle strength in healthy young men. Furthermore, the study determined changes induced in the body composition and hemodynamic parameters after both training exercises to assess the resistance training response. We hypothesize that TRT and FRT would elicit an increase in arterial stiffness.

## Materials and Methods

### Study Design

The current study was designed as a randomized controlled trial. All the participants were randomly assigned using the website^1^ (Social Psychology Network, Connecticut, United States) into either a 6-week TRT group or a FRT group. The trial was prospectively registered at the http://www.chictr.org.cn/ as ChiCTR2100048485. Ethical approval was granted by the Capital University of Physical Education and Sports Ethical Committee. Prior to study initiation, all the participants were informed of the risks and requirements of the training program, and voluntary consent was obtained from all of them. Additionally, the study was conducted while adhering to the CONSORT statement (Schulz et al., 2010).

### Participants

The sample size was estimated based on a similar experimental design (Ozaki et al., 2013). In view of an effect size *f*^2^ = 0.30, with a power of 0.80 and a significance level of 0.05 (Cohen, 1992), the minimum sample size of 24 (12 per group) was found to be adequate using repeated measures within–between interaction (G*Power 3.1; Heinrich Heine, Dusseldorf, Germany). Considering the 15% sample loss, a sample size of 28 was deemed sufficient for the present study. A total of 31 untrained individuals volunteered to participate in this study. All participants were initially screened at the Capital University of Physical Education and Sports in Haidian District, Beijing, China, and were recruited through printed advertisement and by word of mouth. The exclusion criteria were: (1) participants were received regular resistance-type training and aerobic training for at least 6 months before study initiation; (2) those with unnormal blood pressure (>140/90 mmHg); (3) those who regularly smoke or drink alcohol; (4) those with regular intake of any dietary supplement or medication; (5) and those with cardiac arrhythmia, peripheral arterial disease, and sports injury. Two participants were dropped out because of personal reasons, and consequently, 29 participants who met the recruitment conditions were included. All participants were randomly assigned into either the TRT group (*n* = 15) or the FRT group (*n* = 14). The participants in both groups were instructed to avoid attending any extra resistance training or aerobic sports training and to maintain normal eating habit throughout the 6-week training period.

### Intervention

#### Data Collection and Outcome Variables

Data were collected between 10 July 2021 and 30 August 2021. All participants finished baseline data tests in the first week. In case any participant developed sports injury or decided to quit, the intervention was terminated earlier. Primary outcomes were arterial stiffness and muscular strength, whereas the secondary variable was the body composition. Arterial stiffness and body composition were measured by a laboratory administrator, whereas muscular strength was measured by an experienced trainer.

#### TRT and FRT Protocols

The participants in both groups were trained 3 days per week for 6 consecutive weeks. The training program comprised a whole-body workout and five exercises, namely, barbell squat for the lower limb, horizontal bench press for chest muscles, dead lift for back and leg muscles, reverse arm curl for biceps, and seated leg flexion for quadriceps. The participants were asked to perform a warm-up and cool-down exercise, which involved static and dynamic stretching, before and after the training intervention. The participants in the TRT group performed 4–5 sets of 8–12 repetitions at 70% of their 1-repetition maximum (1RM) to volition fatigue, with 1–2 min of rest between the sets. The FRT group performed and maintained same training exercises as the TRT group; however, unstable devices (e.g., BOSU ball, swish balls, and balance discs) were used to increase both core trunk strength and neuromotor and proprioceptive demand in this group. An unstable training condition may not provide the same intensity of overload as TRT under stable conditions (Kibele and Behm, 2009). The participants performed horizontal bench press, dead lift, and barbell squat on the swish ball, balance disc, and BOSU ball, respectively. Additionally, kettlebell swings and Bulgarian split squats were performed on the BOSU ball. Regarding the repetitions of the FRT group, each set training repetition of the FRT group was calculated by the total training volume (70% 1RM lifting weight × repetition) of the TRT group because the training set and rest period were same between the TRT and FRT groups. We controlled the equivalent of the total training volume between the two groups, and the repetition in the FRT group was calculated using the following formula: 70% 1RM lifting weight (kg) × repetition (TRT group)/40%1RM to volition fatigue. Thus, the FRT group performed 4–5 sets of 15–22 repetitions at 40%1RM, with similar rest period between the sets. Table 1 presents the specific training protocol from 1 to 3 weeks and 3 to 6 weeks for both groups. The strength assessments for all the participants were performed again after 3 weeks of intervention to ensure that the participants readjusted training intensities based on strength gains. To minimize any potential diet-induced variability in muscle strength and body composition measurement, the participants in both groups were asked to maintain normal dietary habits and avoid overeating. Additionally, they were asked to refrain from any aerobic exercise throughout the study.

**Table 1:** Resistance training protocol during 6 weeks for TRT group and FRT group.

Variables	Group	S	Rep	TI (KG)	Rest	TV (KG)
**1–3 week**						
Barbell Squat	TRT	4–5	12	70%1RM (81.2)	1-2 min	974.4
Bench Press		4–5	12	70%1RM (52.5)	1-2 min	630
Deadlift		4–5	12	70%1RM (82.5)	1-2 min	990
Reverse Arm Curl		4–5	15	10 kg	1-2 min	150
Leg Flexion		4–5	15	70%1RM (29)	1-2 min	435
						3179.4
Barbell Squat&BOSU	FRT	4–5	16–20	40%1RM (54.8)	1-2 min	876.8–1,096
Bench Press&Swissball		4–5	16–20	40%%1RM (35.5)	1-2 min	568–710
Deadlift&BOSU		4–5	16–20	40%1RM (55.7)	1-2 min	891.2–1,114
Kettlebell Swing&BOSU		4–5	15	20 kg	1-2 min	300
Bulgarian Split Squats &BOSU		4–5	15	16 kg	1-2 min	240
						2,876–3,460
**3–6 week**						
Barbell Squat	TRT	4–5	12	70%1RM (95.6)	1-2 min	1147.2
Bench Press		4–5	12	70%1RM (60)	1-2 min	720
Deadlift		4–5	12	70%1RM (91)	1-2 min	1,092
Reverse Arm Curl		4–5	15	15 kg	1-2 min	225
Leg Flexion		4–5	15	70%1RM (32.5)	1-2 min	487.5
						3671.7
Barbell Squat&BOSU	FRT	4–5	17–21	40%1RM (61.1)	1-2 min	1038.7–1283.1
Bench Press&Swissball		4–5	18–22	40%1RM (36.4)	1-2 min	655.2–800.8
Deadlift&BOSU		4–5	18–22	40%1RM (55.2)	1-2 min	999–1,221
Kettlebell Swing&BOSU		4–5	15	24 kg	1-2 min	360
Bulgarian Split Squats &BOSU		4–5	15	20 kg	1-2 min	300
						3352.9–3964.9

*S, sets; Rep, repetitions, TI, training intensity; TV, training volume, KG, average kilo gram*.

#### Arterial Stiffness Assessment

Studies have reported the cardio-ankle vascular index (CAVI) as a novel, non-invasive, and blood pressure-independent device *VS*-1500AE/AN (Fukuda Denshi, Tokyo, Japan) for evaluating systemic arterial stiffness (Shirai et al., 2011; Koshiba and Maeshima, 2019), which has been successfully applied in healthy young men (Li et al., 2015a). Theoretically, the cardio-ankle vascular index was adjusted independently from blood pressure (Shirai et al., 2011). The degree of arteriosclerosis increases with an increase in the cardio-ankle vascular index, which is related to the cardiovascular disease risk (Shirai et al., 2011). All the participants were in a seated position for 15 min in a quiet, air-conditioned room (23–25°C), according to the method described by Cortez-Cooper et al. (Cortez-Cooper et al., 2003). The participants were asked to fast for at least 6 h and avoid the intake of caffeine and alcohol for 12 h before testing. Before the assessment, a blood pressure cuff was wrapped around the participant’s right upper arm in the supine position, a cardiac sonography microphone on the left parasternal border of the fourth internal space was placed, and then the brachial systolic and diastolic blood pressure were measured using oscillometric method and heart rate was recorded using the electrocardiogram module of VaSera device. Finally, electrocardiogram electrodes were affixed on both femoral and carotid artery. The whole measurement procedure of CAVI and blood pressure conformed strictly to the recommendation (Sun, 2013) and guideline (Pickering et al., 2005), respectively. According to the guideline, a minimum of two readings of blood pressure were taken at intervals of at least 1 min, and the average of two readings will be used to represent the participant’s blood pressure. If there is a >5 mm Hg difference between two readings, additional (1 or 2) readings should be obtained, and then the average of these multiple readings is used. However, as described in the recommendation, CAVI was theoretically independent of changes in blood pressure, and we used the average of those readings as participant’s blood pressure. Both the left and right cardio-ankle vascular indices were automatically calculated using an electrocardiogram, phonocardiogram, and brachial and tibial wave forms (Li et al., 2015a). According to a study, the original formula for calculating the cardio-ankle vascular index from the aforementioned indicators is as follows: CAVI = a × (haPWV)^2^ [2ρ/(*P*sys − *P*dia)] ln(*P*sys/*P*dia) + b, where *P*sys represents the systolic blood pressure, *P*dia is the diastolic blood pressure, haPWV is PWV from the origin of the aorta to tibial artery at the ankle through the femoral artery, ρ represents the blood density, and a and b are the constants to convert the cardio-ankle vascular index to those of Hasegawa’s pulse wave velocity (Asmar, 2017). Prior to normal assessments, four standard cuffs were positioned around the left and right upper arms and ankles, with electrocardiogram leads linked to the wrist and a microphone located on the mid breastbone for phonocardiography. Vascular length (VL) was indirectly assessed from the height of the participants by using the following formula (Li et al., 2015a): VL = 0.77685 × height (cm) − 1.7536. The electrocardiogram on the wrist and the phonocardiogram at the mid breastbone were used to detect the initial notch of pulse wave at the heart and ankle joint. Then, the right and left cardio-ankle vascular indices in each participant were automatically analyzed using the device.

#### One-Repetition Maximal Strength Assessment

Each participant completed the baseline and follow-up 1RM before and after the 6-week training program in the same order: barbell squat, bench press, dead lift, and seated leg flexion. The 1RM tests conformed to the prescription and guidelines of the American College of Sports Medicine (American College of Sports Medicine et al., 2018). The 1RM was measured by gradually increasing the weight lifted until each participant failed to lift the current weight through the whole exercise process. The 1RM test was completed through approximately five trials, with the rest period between each trial being approximately 1–2 min. Firstly, the participant warmed up for 5 min on a paddle ergometer at a perceived exertion level 3 (on the CR 10 Borg scale), followed by familiarization with each testing movement pattern, especially the leg flexion machine for lower limbs. Because the participants were in a seated position, the hip angle was approximately 110°. With verbal encouragement, the participants attempted to perform a concentric of the right leg flexion starting from the extended position of 180° to reach the approximate flexion of 70° against the resistance determined by the loads (kg) selected on the weight back.

#### Handgrip Strength Assessment

Handgrip strength was assessed using a WCS-99.9 Grip-A manual dynamometer (Yilian, Shanghai, China) with a precision of 0.1 kg. The left and right hands of the participants were assessed twice separately, and the mean value was recorded. The relative value of the mean handgrip strength data was transformed by weight as follows: Handgrip strength (N/kg) = measured values × 9.8/body weight. The constant 9.8 illustrates the conversion factor from kg to N.

#### Body Composition and Anthropometric Assessment

The height and weight were measured using a portable stadiometer and an electronic scale before and after the 6-week regular resistance training intervention. Then, the body mass index was calculated according to the following formula: BMI = weight (kg)/height (m)^2^. The body composition, including body fat and lean body mass, was assessed using bioelectrical impedance (Tanita MC-980MA, Tokyo, Japan), with the participants wearing light clothes and no footwear. Body composition and anthropometric measurements of the participants who fasted overnight (>8 h) were simultaneously assessed before and after the intervention. This detection method can measure changes in the human body composition accurately and with high validity (Miyatani et al., 2012).

### Statistical Analysis

Statistical analyses were performed using SPSS version 22.0 Windows (SPSS, Inc., Chicago, IL, United States). All baseline and post-intervention data are presented as mean ± standard deviation (SD) and were initially confirmed to be normally distributed by using Bartlett’s and Levene’s tests. Homogeneity of variance was tested before further statistical analyses. Thus, no transformation was required.

Independent samples *t*-test and paired *t*-test were used to examine difference between the two groups at baseline and the differences within groups following the intervention, respectively. Between-group differences following the intervention were analyzed using a two-way repeated measures analysis of variance [time (pre vs. post) — group (TRT vs. FRT)]. When the significant interactions were found, the independent samples *t*-test was applied to determine inter-group differences. A value of p of <0.05 was considered statistically significant.

For main parameters, mean difference in change (pre – post-interventions) for the two groups are reported, and the respective effect sizes calculated as partial eta square converted to Cohens *d*, being classified as “small” from 0 to 0.2 “medium” from 0.2 to 0.8, and “large” if higher than 0.8 (Cohen, 1992).

## Results

### Participants

**Figure 1:**
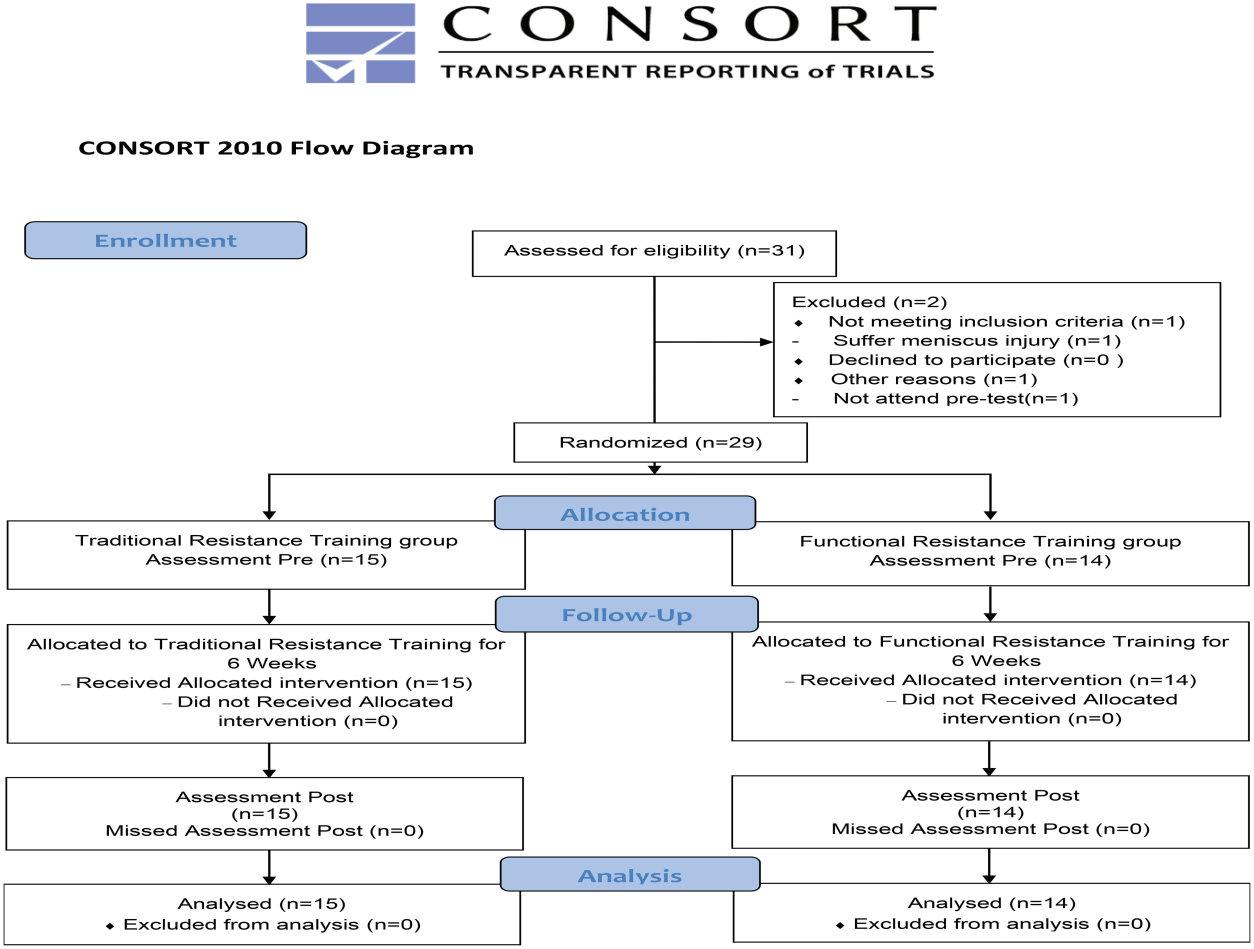
CONSORT flow diagram of participants’ training schedule through the study.

Of 31 young men assessed for eligibility, 29 participants were selected in this study, as shown in the flow diagram (Figure 1). Two men were dropped out because of personal reasons, mainly meniscus injury and non-participation in the pre-test. All participants were randomly assigned into the TRT group (*n* = 15) and FRT group (*n* = 14). Table 2 presents the main characteristics of the participants at baseline. No significant differences between the groups were observed in terms of age, height, body weight, and body mass index. Additionally, all the participants in both groups adhered to the 18 scheduled training sessions during the intervention period. No training-related injuries were observed, and none of the participants quit the study.

**Table 2:** Group characteristics at baseline as Mean ± SD.

	TRT group (*n* = 15)	FRT group (*n* = 14)	Group difference *p*-value
Age(y)	22.1 ± 2.9	20.9 ± 2.7	0.261
Height(cm)	176.6 ± 5.4	176.7 ± 6.0	0.958
Body weight(kg)	77.9 ± 11.6	73.4 ± 10.2	0.268
Body mass index(kg/m^2^)	24.9 ± 3.1	23.4 ± 2.6	0.168

*TRT, traditional resistance training group; FRT, functional resistance training group*.

### Arterial Stiffness

Regarding arterial stiffness outcomes, training decreased the right cardio-ankle vascular index (TRT, −0.51 and FRT, −0.53; main effect of time *p* = 0.03) and left cardio-ankle vascular index (TRT, −0.44 and FRT, −0.61, main effect of time *p* = 0.04); however, no significant difference was found between the groups, and the effect sizes indicated small to medium effects in favor of the FRT group (Cohen *d* = 0.04 and 0.28 for the right cardio-ankle vascular index and left cardio-ankle vascular index, respectively). As described in Table 3, no significant difference was observed in any of the hemodynamic characteristics.

**Table 3:** Change in arterial stiffness as mean difference, statistical test of group difference and effect sizes as Cohen *d*.

	TRT group (*n* = 15)			FRT group (*n* = 14)			Group *p*-value	Effect sizes
	Pre	Post	Mean differ	Pre	Post	Mean differ		
b SBP (mmHg)	126.8 ± 11.9	123.7 ± 12.0	−3.07	128.3 ± 7.2	125.3 ± 9.1	−3.00	0.577	0.15
b DBP (mmHg)	71.9 ± 6.2	73.2 ± 5.3	1.23	74.3 ± 6.0	70.5 ± 6.2	−3.79	0.832	0.46
Mean BP (mmHg)	90.2 ± 7.6	90.0 ± 6.3	−0.20	92.3 ± 5.4	88.8 ± 6.0	−3.52	0.811	0.20
PP (mmHg)	54.9 ± 8.2	50.6 ± 11.0	−4.30	54.0 ± 7.5	54.8 ± 8.8	0.79	0.518	0.42
Heart rate (bmp)	59.0 ± 10.0	59.1 ± 9.9	0.08	64.2 ± 11.3	61.1 ± 11.1	−3.07	0.261	0.25
R-CAVI	6.2 ± 0.6	5.7 ± 0.6^*^	−0.51	5.9 ± 0.5	5.3 ± 0.6^*^	−0.53	0.042	0.04
L-CAVI	6.2 ± 0.6	5.7 ± 0.6	−0.44	6.0 ± 0.6	5.4 ± 0.6^*^	−0.61	0.072	0.28

*b, brachial; SBP, systolic blood pressure; DBP, diastolic blood pressure; BP, blood pressure; PP, pulse pressure; R-CAVI, right cardio-ankle vascular index; L-CAVI, left cardio-ankle vascular index; TRT, traditional resistance training group; FRT, functional resistance training group; differ, difference;* ^*^p < 0.05, main effect of time.

### Muscle Strength Measurements

Table 4 presents the results of the 1RM strength assessed for the two study groups before and after resistance training. All 1RM tests indicated a significant main effect of time (*p* < 0.01), and both groups exhibited enhanced dynamic strength in all the exercise analyzed. However, the maximal muscular strength was not found to differ between training protocols, and the effect sizes indicated small effects in favor of FRT (Cohen *d* = 0.20, 0.02, and 0.04 for bench squat, bench press, and dead lift, respectively).

**Table 4:** Change in muscle strength variables and handgrip strength as mean difference, statistical test of group difference and effect sizes as Cohen d.

	TRT group (*n* = 15)			FRT group (*n* = 14)			Group *p*-value	Effect sizes
	Pre	Post	Mean differ	Pre	Post	Mean differ		
BS (kg)	116.0 ± 19.9	147.5 ± 15.1^**^	31.50	114.3 ± 16.0	148.9 ± 15.1^**^	34.64	0.979	0.20
BP (kg)	75.0 ± 9.8	89.3 ± 10.9^**^	14.33	71.4 ± 10.3	85.9 ± 10.4^**^	14.46	0.343	0.02
DL (kg)	118.7 ± 21.3	139.0 ± 16.7^**^	20.33	110.0 ± 25.4	129.8 ± 18.4^**^	19.82	0.234	0.04
R-LF (kg)	43 ± 6.5	50.7 ± 8.0^**^	7.67	39.3 ± 6.8	49.6 ± 8.9^**^	10.36	0.385	0.58
M Hg (kg)	41.8 ± 6.0	45.9 ± 5.2^**^	4.04	40.5 ± 8.4	45.8 ± 8.4^**^	4.94	0.728	0.32
M Hg (N/kg)	5.4 ± 1.0	5.9 ± 0.8^**^	0.59	5.4 ± 0.8	6.1 ± 0.8^**^	0.70	0.720	0.28

BS, barbell squat; BP, bench press; DL, dead lift; R-LF, right leg flexion; M Hg, mean handgrip; TRT, traditional resistance training group; FRT, functional resistance training group; differ, difference, ^**^p < 0.01, main effect of time.

The main time effect was detected for mean handgrip strength (*p* < 0.01), whereas no significant differences were found in mean handgrip and relative mean handgrip between the groups, and a medium effect size (Cohen *d* = 0.32 and 0.28 for mean and relative mean handgrip respectively, Table 4) was demonstrated in favor of FRT.

### Body Compositions Measurements

The descriptive data for body composition measurements are presented in Table 5. The body fat decreased significantly (*p* < 0.01) and lean body mass increased significantly (*p* < 0.05) in both training groups after 6 weeks, with no difference between the training modes (main effect of time, *p* < 0.05). However, a significant interaction between time and group was detected for body weight and BMI (*p* < 0.05) when the within-group difference was analyzed only by TRT and FRT (for body weight: TRT *p* < 0.05, FRT *p* = 0.757; for BMI: TRT *p* < 0.05, FRT *p* = 0.690); however, no differences were observed between the groups (pre *p* = 0.270, post *p* = 0.423 for body weight and pre *p* = 0.168, post *p* = 0.304 for BMI).

**Table 5:** Body composition measurements change from pre to posttest.

	TRT group (*n* = 15)		FRT group (*n* = 14)	
	Pre	Post	Pre	Post
Body weight(kg)	77.9 ± 11.6	76.4 ± 9.6^#^	73.4 ± 10.2	73.5 ± 9.5
BMI (kg/m^2^)	24.9 ± 3.1	24.4 ± 2.5^#^	23.4 ± 2.6	23.5 ± 2.4
Body fat (%)	18.8 ± 5.8	16.5 ± 5.3^**^	16.7 ± 4.6	15.3 ± 4.7^**^
Lean body mass(kg)	59.5 ± 5.4	60.4 ± 4.9^*^	57.7 ± 6.7	58.8 ± 5.9^*^

BMI, body mass index, TRT, traditional resistance training group, FRT, functional resistance training group, ^*^p < 0.05, ^**^p < 0.01, main effect of time, ^#^p < 0.05 from pre-intervention.

## Discussion

The present study was designed to investigate the effects of an equal volume of 6-week supervised traditional and functional resistance training programs on the maximal strength and arterial stiffness in healthy young men. To the best of our knowledge, none of the studies have investigated the maximal strength and arterial stiffness following short-term functional resistance pattern in healthy men. In this study, both resistance training patterns were found to decrease the right and left cardio-ankle vascular indices after 6 weeks of intervention compared with those before the intervention; however, the between-group differences were found to be non-significant. This study also demonstrated that the 6-week FRT is equally effective as TRT in improving muscle strength and achieving positive effects on the body composition. In a study, Ozaki et al. (2013) reported that carotid arterial stiffness decreased and 1RM strength increased following 6-week high-intensity resistance training in young men. This finding is concurrent with that of our study. Therefore, our results indicated that both traditional and functional resistance training programs may be an effective exercise pattern for improving systematic arterial stiffness and muscular strength under the equivalent resistance training volume.

TRT is effective for the enhancement of functional performance and prevention of sarcopenia (Williams et al., 2007). However, studies conducted on different types of resistance training in individuals have indicated contradictory outcomes regarding the TRT-induced changes in arterial stiffness. Miyachi et al. (Miyachi et al., 2004) demonstrated that 4 months of regular whole-body high-intensity resistance training (6 exercise, 3 sets per session) decreased approximately 20% of cardio arterial compliance in young men. Cortez-Cooper et al. (2005) also reported that arterial stiffness and wave reflection increased following 11-week high-intensity resistance training (12 exercise, up to 6 sets per session) in young women. By contrast, Werner et al. (Werner et al., 2019) observed that the arterial stiffness indices exhibited no changes following 12 weeks of high-intensity and high-volume resistance training (9 exercise, 2–3 sets, and 3–4 sets, respectively) in young men. The findings of this study are in agreement with the results of Au et al. (2017), who found a decrease in central arterial stiffness following 12 weeks of total body resistance training with different loads (heavier, 75–90% 1RM; 5 exercise, 3 sets vs. lighter, 30–50% 1RM, 5 exercise 3 sets) in health young men. In the current study, 6 weeks of whole-body traditional and functional resistance training with different loads (70%1RM, 5 exercise, 4–5 sets vs. 40%1RM, 5 exercise, 4–5 sets) decreased the right cardio-ankle vascular index by 7.4 and 8.2%, respectively, whereas the left cardio-ankle vascular index decreased to 6.2 and 9.4% in the TRT group and FRT group, respectively. However, no significant difference was observed in the delta change of cardio-ankle vascular index between the groups, Thus, from the perspective of reducing systemic arterial stiffness, we believe that both training protocols were equally effective in improving the cardio-ankle vascular index. However, to our knowledge, this study is the first to measure the cardio-ankle vascular index following the short-term resistance training in young men and to compare the effects of the traditional and functional resistance training, as the differences in training volumes in the current and previous studies that may not explain the adaptations of arterial stiffness by traditional and functional resistance training.

The effects of resistance training on the cardio-ankle vascular index in adults have been demonstrated in only two long-term studies and one acute study. Yasuda et al. used the blood flow-restricted low-intensity resistance training and exhibited an increase in the maximum muscle strength; however, the training did not appear to influence arterial stiffness (Yasuda et al., 2014). Conversely, Li et al. exhibited that the cardio-ankle vascular index decreased in young people for at least 60 min after acute resistance training (70% 1RM of lower limb vs. whole-body; 6 exercise, 4 sets; Li et al., 2015a). Similarly, a recent study reported an improvement in the cardio-ankle vascular index following 12-week resistance training (50–60% 1RM, 11 exercise, 1–3 sets) (Gholami et al., 2021). These findings reflect the beneficial effects of resistance training on elastic properties of systemic arteries. Furthermore, these findings suggest that acute or chronic resistance training may exert beneficial effects on the vascular function, and our data suggest the traditional and functional resistance training exert no adverse effect on the arterial function, consistent with the findings of other studies (Miyachi et al., 2004; Cortez-Cooper et al., 2005).

The improved cardio-ankle vascular index response following chronic resistance training can be attributed to many factors. The physiological mechanism of the reduced arterial stiffness following resistance training is not well-understood yet; however, the dilatation of muscular arteries appears to be the main mechanism. Evidence suggests that exercise-induced reduction of arterial stiffness is accompanied by the muscular arterial distension responses and the increased blood flow (Munir et al., 2008). In a previous study, the increased use of vasodilator agents (e.g., nitroglycerin) may have caused arterial stiffness changes comparable to increasing training intensity, but none of the participants in this study took any supplements or medication, which is indicated that resistance training provoked similar adaptations of arterial stiffness (Li et al., 2015a). In addition, high shear stress is responsible for the increase in the nitric oxide concentration during strenuous exercise, and it leads to an improvement in vasodilatory capacity and hemodynamic parameters (Munir et al., 2008). Munir et al. (2008) reported that the vascular smooth muscle tone decreased with vasodilation, because of which arterial wall stress was transferred from harder collagen fibers to more elastin fibers, making the arterial wall more flexible. Although the present study observed no significant changes in lean body mass between the two groups, the surface area of the muscular arteries might be greater than before in both groups. Hence, future research will be needed to determine the effect of resistance training type induced difference in surface area of the muscular arteries on arterial stiffness.

As mentioned previously, the most notable difference between the resistance training program used in the present study and other studies is the strictly controlled training volume of the two groups during the training process in the present study. Despite differences in the training intensity and condition, the increased muscular strength and lean body mass exhibited no difference between both groups in our study. Several studies have employed fewer exercises (Casey et al., 2007; Okamoto et al., 2013), more sets (Cortez-Cooper et al., 2005), or lower training frequencies (Okamoto et al., 2011), all of which are not favorable for ideal muscular strength and lean body mass gains. Probably, skeletal muscle adaptations (strength and muscle mass) were determined in response to equal-volume resistance training with divergent training strategies. Kubo et al. (2021) reported that the increase in muscle volume was similar among the three training protocols, namely, 4RM, 8RM, and 12RM, under equal training volume. Similarly, Colquhoun et al. (Arazi et al., 2021) found that three resistance training sessions per week provided similar increase in muscle strength and fat-free mass compared with six sessions per week under equal-volume condition. Hence, we assume that the muscle adaptation status could be same in response to TRT and FRT protocols under an equal volume.

Two modalities of resistance training, which differed in terms of surface condition and intensity, were considered in the present study. The 1RM strength parameters of the upper and lower limb and muscle mass observed in our study are consistent with those indicated in similar studies (Soligon et al., 2020; Brown et al., 2021; Hamid et al., 2022). Kibele and Behm (Maté-Muñoz et al., 2014) reported that the traditional resistance training characteristics were to perform higher overload weights than in functional resistance training, which could also obtain similar muscle strength responses with the use of lower resistive load under unstable condition. The comparison of data between the two groups exhibited that despite forces were applied without overload to the upper and lower muscles in the FRT group when using an instability device for training, strength enhancements were probably related to the increase in trunk and lower muscle activation (Anderson and Behm, 2005), sympathetic transmission, and recruitment of motor neurons, which may endorse intramuscular and intermuscular coordination and cooperation (Asanuma and Pavlides, 1997) and make the agonist muscle activation more economic, thereby enhancing the strength performances.

Additionally, the greatest strength enhancements were observed in the lower limbs (e.g., Barbel Squat, 29.8 and 31.6% increase for the TRT and FRT groups, respectively) because the selected motor patterns in both groups were mainly standing and lower limbs, such as the Bulgarian split squats. Peter (2013) reported that the center of gravity tends to swing as the body moves along a vertical axis, increasing the degree of lower limb instability, which could be conducive to trunk and lower limb muscle activation (Anderson and Behm, 2005) and intramuscular and intermuscular coordination.

Regarding the body composition, our results indicated significant changes in body fat percentage and lean body mass in both groups, but the difference between the groups was non-significant in terms of these parameters. This result is consistent with that of a previous study (de Resende-Neto et al., 2019a). Several studies have demonstrated the efficiency of resistance training in neuromuscular and metabolic stimulation to endorse tissue structure changes, such as reduced adipose and increased muscle tissues (Hunter et al., 2013; Marín-Cascales et al., 2018). Another interesting result of the present study is that the body weight and BMI decreased significantly in the TRT group but not in the FRT group, which does not seem logical. Previous studies on the metabolic response to FRT found an average caloric expenditure of approximately 10.1 kcal for one-minute functional resistance training, which is higher than the expenditure of 5–9 kcal/min reported in studies examining traditional resistance exercise (Bloomer, 2005; Lagally et al., 2009). Probably, the reason should be that the TRT group in the present study performed a high-intensity workout (at least 4–5 sets of 12 repetitions per training) until exhaustion and produced more energy consumption. Thus, we assume that the caloric expenditure of the TRT protocol is somewhat higher than that of the FRT protocol (Tomljanović et al., 2011).

The present study has certain limitations. We measured the right and left cardio-ankle vascular indices in the two groups; however, no significant decrease was observed in L-CAVI in the TRT group. We hypothesize that the decrease in arterial stiffness could be related to the participants’ dominant side because all the participants in this study were right-dominant, and future research should explore the effect of dominant side on arterial stiffness. Furthermore, the study participants were limited to healthy young men with normal arterial wall and function; thus, the outcomes could not be generalized to women or individuals with other chronic diseases, such as diabetes, heart disease, and hypertension. Moreover, the 6-week intervention duration may not have been long enough to cause significant changes in muscular strength and arterial stiffness between the two groups, and due to the lack of a control group, the results between the two groups only exhibited the main effects of time without the time × group interaction effect. Therefore, future studies with a larger sample size and different subject types, ages, and intervention periods are required to determine the excellent resistance training pattern beneficial for health.

## Conclusion

The study demonstrated that the effects of 6-week TRT and FRT on arterial stiffness and muscular strength in healthy young men do not differ significantly. These results could be attributed to muscular arterial distension responses. Although previous studies have shown unfavorable effects of resistance training on arterial stiffness, our results suggest beneficial alternations on the vasculature with both traditional and functional resistance training protocols in young men. These results support the beneficial role of resistance training in improving the cardiovascular health function.

## Data Availability Statement

The raw data supporting the conclusions of this article will be made available by the authors, without undue reservation.

## Ethics Statement

The studies involving human participants were reviewed and approved by the Capital University of Physical Education and Sports in Haidian District, Beijing, China after institutional ethics clearance. Written informed consent has been obtained from the patient(s) to publish this paper. The patients/participants provided their written informed consent to participate in this study.

## Author Contributions

CZ and SB contributed to the conceptualization and design of the study and reviewed the manuscript. CZ, QL, and LZ contributed to data collection. SB submitted the methodology. CZ conducted the formal analysis and wrote the first draft of the manuscript. All authors have read and agreed to the published version of the manuscript.

## Conflict of Interest

The authors declare that the research was conducted in the absence of any commercial or financial relationships that could be construed as a potential conflict of interest.

## Publisher’s Note

All claims expressed in this article are solely those of the authors and do not necessarily represent those of their affiliated organizations, or those of the publisher, the editors and the reviewers. Any product that may be evaluated in this article, or claim that may be made by its manufacturer, is not guaranteed or endorsed by the publisher.

## Footnotes

^1^ http://randmizer.org/
